# Unraveling the Peculiar Features of Mitochondrial Metabolism and Dynamics in Prostate Cancer

**DOI:** 10.3390/cancers15041192

**Published:** 2023-02-13

**Authors:** Fabrizio Fontana, Martina Anselmi, Patrizia Limonta

**Affiliations:** Department of Pharmacological and Biomolecular Sciences, University of Milan, 20133 Milan, Italy

**Keywords:** prostate cancer, mitochondrial metabolism, OXPHOS, Warburg effect, mitochondrial dynamics

## Abstract

**Simple Summary:**

Mitochondria are organelles involved in different biological processes, including tumorigenesis. Accumulating evidence strongly supports that, despite the presence of an active glycolytic pathway (the Warburg effect), cancer cells undergo a mitochondrial metabolic rewiring towards the OXPHOS pathway, leading to the production of high levels of ATP to sustain their uncontrolled proliferation and aggressive behavior. Alterations of the mitochondrial structural dynamics (biogenesis, fusion, fission, mitophagy) are also involved in cancer growth and progression. The mechanisms underlying this mitochondrial plasticity, now considered as a hallmark of cancer, were shown to occur with specific features in different cancer cell types and contexts. In this review, we provide an incisive description of the peculiar metabolic rewiring and structural dynamics occurring in prostate cancer cells during the different phases of their transformation from healthy cells to early- and late-stage cancer cells. We also address the role of these mitochondrial dynamics as effective targets of therapeutic approaches.

**Abstract:**

Prostate cancer (PCa) is the second leading cause of cancer deaths among men in Western countries. Mitochondria, the “powerhouse” of cells, undergo distinctive metabolic and structural dynamics in different types of cancer. PCa cells experience peculiar metabolic changes during their progression from normal epithelial cells to early-stage and, progressively, to late-stage cancer cells. Specifically, healthy cells display a truncated tricarboxylic acid (TCA) cycle and inefficient oxidative phosphorylation (OXPHOS) due to the high accumulation of zinc that impairs the activity of m-aconitase, the enzyme of the TCA cycle responsible for the oxidation of citrate. During the early phase of cancer development, intracellular zinc levels decrease leading to the reactivation of m-aconitase, TCA cycle and OXPHOS. PCa cells change their metabolic features again when progressing to the late stage of cancer. In particular, the Warburg effect was consistently shown to be the main metabolic feature of late-stage PCa cells. However, accumulating evidence sustains that both the TCA cycle and the OXPHOS pathway are still present and active in these cells. The androgen receptor axis as well as mutations in mitochondrial genes involved in metabolic rewiring were shown to play a key role in PCa cell metabolic reprogramming. Mitochondrial structural dynamics, such as biogenesis, fusion/fission and mitophagy, were also observed in PCa cells. In this review, we focus on the mitochondrial metabolic and structural dynamics occurring in PCa during tumor development and progression; their role as effective molecular targets for novel therapeutic strategies in PCa patients is also discussed.

## 1. Introduction

Mitochondria are organelles involved in different cellular processes, including cell proliferation and intrinsic apoptosis, redox and Ca^2+^ homeostasis as well as cell stemness. They are also known as the master producers of ATP, being deeply involved in cellular energy metabolism; in addition, being dynamic organelles, they often undergo structural changes, including biogenesis, fusion/fission and mitophagy. Specifically, it is now well recognized that mitochondria undergo complex functional and structural dynamics in cancer cells during the different phases of tumor growth and progression. 

### 1.1. Mitochondrial Metabolism in Cancer Cells

It is now recognized that cancer cells, growing in a hypoxic and hyponutrient microenvironment, are forced to adapt their metabolism (“metabolic reprogramming”) to obtain the required amount of biomass and energy to sustain their uncontrolled proliferation and aggressive behavior. 

According to the theory proposed by Otto Warburg in the 1920s (named the Warburg effect), cancer cells are characterized by high rates of glucose uptake and preferentially metabolize it through the glycolytic pathway, even in the presence of adequate amounts of oxygen (aerobic glycolysis) and functional mitochondria [1,2]. Although a small amount of ATP per mole of glucose is produced through glycolysis, it is believed that this metabolic process rapidly produces high levels of metabolites to sustain the biosynthesis of the molecules (i.e., amino acids, fatty acids and nucleotides) required for cancer cell growth and division [3,4,5,6,7,8]. Moreover, the high levels of lactate produced at the end of glycolysis by lactate dehydrogenase (LDH) are secreted by cancer cells to generate an acidic tumor microenvironment promoting their transition to the most aggressive (i.e., migratory, invasive) phenotype and affecting the immune microenvironment to induce an immune tolerant condition [9,10]. 

Despite the presence of an active glycolytic pathway, several recent data strongly support the coexistence of functional mitochondria in cancer cells, even in the metastatic phase [6,11,12,13,14,15,16,17,18,19,20]. Mitochondria, known as the “powerhouse of the cell”, are deeply involved in the cellular metabolic dynamics, being the major intracellular producers of ATP through the oxidative phosphorylation (OXPHOS) pathway; these organelles are also the “venue” where the tricarboxylic acid (TCA) cycle takes place to provide the most building blocks for the synthesis of biomolecules. A high level of fatty acid β-oxidation also occurs in mitochondria to sustain the production of citrate. Moreover, glutaminolysis is activated to convert glutamine into intermediates for the synthesis of amino acids and nucleotides, as well as into glutamate to fuel the TCA cycle. Together, these metabolic pathways are necessary for cell anabolism to trigger cell proliferation and metastasis in cancers [8,20,21,22,23,24,25,26,27,28,29,30]. 

A metabolic remodeling based on a shift towards the OXPHOS machinery has also been shown to be involved in the development of drug resistance in tumor cells [31,32,33,34] and to occur in the subpopulation of cancer stem cells (CSCs), known to play a key role in tumor relapse, to provide sufficient amounts of energy and metabolites for their self-renewal and evasion from cell death induced by anticancer drugs [35,36,37,38,39,40,41,42,43]. 

Cancer cell metabolic plasticity has also been reported to be regulated by neighboring cells in the tumor microenvironment through both mechanical and chemical factors [18,44]. For instance, it has been shown that cancer-associated fibroblasts (CAFs) secrete lactate that is taken up by tumor cells to trigger their metabolic switch towards the OXPHOS energy-producing pathway and reactive oxygen species (ROS) generation [45,46,47,48]. 

The key role played by mitochondrial metabolism in cancer is strongly supported by the observation that different compounds were shown to exert their anticancer activity by targeting the oxidative phosphorylation pathways [42,49,50,51,52,53,54,55,56]. 

In addition to their central role in energy production, these organelles are also involved in different biological processes, such as ROS production and signaling, Ca^2+^ homeostasis and apoptosis [57,58,59,60,61]. ROS are generated as by-products of an impaired activity of the mitochondrial electron transport chain (ETC), specifically of respiratory complexes I, II and III, leading to an excessive production of electrons that are directly transferred to oxygen-producing superoxides [62]. It is now clear that the effects of ROS on oxidative cell signaling depend on the specific type of generated ROS, their localization and, in particular, their concentration [63,64,65,66,67]. In cancer cells, low or moderate levels of ROS were shown to be associated with activated mitogenic pathways, supporting cell growth and metastatic behavior, and with a survival advantage to stress-inducing stimuli from the microenvironment [57,64,68,69]. On the other hand, high levels of ROS were widely reported to induce DNA damage and to trigger pro-death mechanisms (i.e., apoptosis, paraptosis, necroptosis) [57,70,71] as well as to mediate the activity of several anticancer drugs [72]. ROS overproduction is frequently observed as a consequence of increased endoplasmic reticulum (ER)-derived Ca^2+^ levels in mitochondria. Ca^2+^ overload and ROS-associated mitochondrial dysfunctions have been reported to be linked to the induction of apoptosis and paraptosis [54,73,74,75,76]. 

### 1.2. Mitochondrial Dynamics in Cancer Cells

Mitochondria are also highly dynamic organelles undergoing changes in number and structure through different processes such as biogenesis, fusion, fission (fragmentation) and mitophagy (removal of impaired mitochondria). A balance between these processes is required for the maintenance of homeostasis in healthy cells; on the other hand, adaptations of mitochondrial dynamics have been widely reported in cells undergoing metabolic and stressful conditions (i.e., glucose starvation, hypoxia), such as cancer cells [8,77,78,79,80,81,82]. 

Mitochondrial biogenesis is the generation of new organelles from pre-existing ones. In cancer cells, an increase in mitochondrial mass is induced by a variety of stressful signals and has been found to correlate with cell growth, invasiveness, metastasis and drug resistance [83,84]. The master regulator of mitochondrial biogenesis is peroxisome proliferator-activated receptor gamma coactivator 1α (PGC1α) which cooperates with different transcription factors to increase the expression of the mitochondrial transcription factor (TFAM), the final effector of the increase of mitochondrial mass [85]. PGC1α, activated by phosphorylation by the energy sensor adenosine monophosphate-activated protein kinase (AMPK) and by deacetylation by silent information regulator 1 (SIRT1), also triggers the transcription of both nuclear and mitochondrial genes, leading to increased mitochondrial mass, OXPHOS activity and ATP production [86,87,88]. Mitochondrial biogenesis has been shown to mediate the ability of CSCs to overcome antitumor therapies [89,90,91]. 

An imbalance in the mitochondrial fusion/fission leads to peculiar changes of the morphological features of these organelles (interconnected vs. fragmented). GTPases belonging to the dynamin family play a pivotal role in mediating both of these processes [16,60,79,82]. Mitochondrial fusion is the physical merging of the outer membranes (OMM) and the inner membranes (IMM) of distinct mitochondria and depends on GTP hydrolysis. This process foresees the activity of three GTPases, mitofusin (MFN) 1 and 2 on the OMM and optic atrophy protein 1 (OPA1) on the IMM. MFN 1 and 2 interact to induce the formation of strict connections between adjacent mitochondria, leading to the fusion of the OMM. Then, OPA1 interacts with MFNs forming intermembrane protein complexes, thus coupling the fusion of OMMs with IMMs [92,93,94,95]. Mitochondrial fission is a multi-step process allowing the division of one mitochondrion, leading to the formation of new organelles. The key protein involved in this process is dynamin-related protein 1 (DRP1), a cytosolic GTPase. Endoplasmic reticulum (ER) membranes get in touch with mitochondria allowing a Ca^2+^ flux from the ER into the mitochondria, thus favoring actin polymerization and inner mitochondrial membrane constriction at this site. At the level of ER-mitochondria contact sites, different proteins such as mitochondrial dynamics 49 and 51 (MID49 and MID51), MFF and mitochondrial fission 1 (FIS1) proteins, identified as DRP1 receptors, are also located. DRP1 binds to these proteins to encircle and shrink the mitochondria, finally leading to their fission. Accumulating evidence demonstrates that an imbalance in these mitochondrial dynamics occurs in different types of cancer [79,96,97,98,99]. 

Mitophagy is the process by which damaged mitochondria are selectively removed, thus representing a key mechanism of mitochondrial quantity and quality control. This process foresees the engulfment of mitochondria in autophagosomes by which the organelles are transferred to lysosomes where they are degraded [100]. The PTEN-induced serine/threonine kinase 1 (PINK1)/Parkin (an E3 ubiquitin ligase) pathway plays a pivotal role in detecting dysfunctional mitochondria and in activating mechanisms leading to their removal and replacement. In the presence of damaged mitochondria, PINK1 localizes at the OMM level where it phosphorylates (i.e., activates) Parkin, which triggers the ubiquitination of different target proteins (mitophagy receptors), such as FIS1, MFN1/2, Miro and voltage-dependent anion channel-1 (VDAC1). These proteins, in turn, recruit the autophagic adaptors p62 and optineurin (OPTN) and selectively interact with the autophagosomal marker LC3 that mediates mitochondria engulfment into autophagosomes, thus, ultimately, leading to their degradation at the lysosomal level [101,102,103]. Dysregulations of the mitochondrial mitophagic process have been reported to occur in different diseases, including cancer [102,104,105,106]. Last but not least, it has been reported that changes in the mitochondrial structural dynamics together with mtDNA damage (i.e., mutations, altered copy number and transcription) correlate with changes in mitochondrial respiration and ATP production in cancer cells. 

## 2. Mitochondrial Metabolism and Dynamics in PCa

Prostate cancer (PCa) represents the most common malignancy among men and the second leading cause of cancer deaths in developed countries [107]. Androgen-deprivation therapy (ADT), based on gonadotropin-releasing hormone (GnRH) agonists and antagonists, either alone or in combination with androgen receptor antagonists (enzalutamide, apalutamide, darolutamide), still remains the most common treatment for androgen-dependent PCa patients [108,109,110,111,112,113,114]. However, PCa often progresses towards the castration-resistant phase (CRPC), a condition characterized by the acquisition of a more aggressive and metastatic behavior even in the absence of circulating androgens [115,116]. The standard treatment of CRPC patients is presently based on chemotherapy (docetaxel) either alone or in combination with GnRH analogs, antiandrogens or inhibitors of androgen synthesis (abiraterone) [117,118,119,120,121,122,123,124,125,126,127]. Novel therapeutic strategies, such as immune check-point inhibitors or CAR-T approaches, are under investigation [128,129,130,131]. The elucidation of novel molecular hallmarks of tumor progression (i.e., mitochondrial metabolism and dynamics) will likely pave the way for the development of novel therapeutic approaches for PCa patients. Based on the above considerations, as well as on the progressively accumulating data in the literature, in this review we aim to highlight and discuss the recent findings on the involvement of mitochondrial functional reprogramming and structural dynamics in PCa, specifically in its CRPC stage; we also address the impact of these mechanisms as molecular targets of novel and effective antitumor strategies for this aggressive disease.

### 2.1. Mitochondrial Metabolism

Metabolic reprogramming is a well-recognized hallmark of cancer, enabling cancer cells to acquire properties that support cell survival, proliferation and acquisition of aggressive (invasive, metastatic) features. However, peculiar molecular mechanisms of this metabolic rewiring have been reported to occur in different types of tumor cells, and this metabolic heterogeneity confers differences in their proliferative/metastatic potential. In prostate epithelial cells, distinctive changes of cell metabolism have been highlighted during the different phases of their conversion from healthy cells to early-stage and, progressively, to late-stage cancer cells [31,132,133,134,135].

#### 2.1.1. Metabolic Rewiring

Healthy epithelial cells, located in the peripheral zone of the prostate, exhibit a peculiar metabolic programming aimed at producing and secreting citrate into the prostatic fluid, one of the most relevant functions of this gland [136]. In most mammalian cells, pyruvate, the end product of the glycolytic pathway, is transported into the mitochondria where it is decarboxylated to acetyl-CoA. Acetyl-CoA subsequently binds to oxaloacetic acid to form citrate that enters the TCA cycle. Citrate is then converted into its isomer isocitrate that is further oxidized into the TCA cycle for the progression to OXPHOS and ATP production; mitochondrial aconitase (m-aconitase) is the ROS-sensitive key enzyme responsible for the citrate-isocitrate conversion. In normal prostate epithelial cells, m-aconitase activity is inhibited, resulting in the impairment of citrate oxidation followed by its accumulation and secretion [137]. The inhibition of m-aconitase strictly correlates with the ability of these cells to accumulate zinc, due to their elevated expression of its transporter ZIP1; high intramitochondrial zinc levels increase ROS generation, leading to the inhibition of m-aconitase activity and resulting in a truncated TCA cycle [138]. As a consequence, the healthy “zinc-accumulating, citrate-producing” epithelial cells are characterized by an inefficient OXPHOS which is compensated by an increased glycolytic pathway to support citrate production [139]. High zinc levels were also found to be associated with a mitochondrial apoptotic phenotype mediated by the release of cytochrome *c* and caspase activation [140]. 

On other hand, it is now well established that prostate epithelial cells undergo a peculiar metabolic rewiring during the early phases of cancer development. Specifically, elevated levels of the TCA cycle enzymes and intermediate metabolites could be detected in prostate cancer tissues in comparison to adjacent normal tissues [141,142,143]. Intracellular zinc levels were found to be significantly reduced in PCa cells, thus leading to the reactivation of m-aconitase, citrate oxidation, TCA cycle pathways and oxidative phosphorylation [144]. This reduction was shown to be related to a decreased expression of zinc transporters, such as ZIP1 and 3 [145], mediated by the hypermethylation of their gene promoters [146]. The low levels of zinc also allow cancer cells to avoid apoptosis; actually, it has been reported that zinc treatments trigger cell death and promote chemosensitivity in PCa cells [147]. In line with these observations, very low zinc levels were observed in PCa tissues [148]. Taken together, these data support that the transformation of prostate epithelial cells into their tumoral phenotype is associated with an efficient reactivation of the TCA cycle/OXPHOS metabolic pathway to meet their increased energy and metabolite demand [149]. 

Interestingly, prostate epithelial cells seem to possess a markedly metabolic plasticity by changing their mitochondrial metabolic features again when progressing from the early-stage towards the late-stage (i.e., metastasis) of cancer, even in the presence of low intracellular zinc levels. Specifically, the Warburg effect (i.e., increased glycolytic activity) has been proposed as the prominent metabolic feature of metastatic prostate tumors [149,150]. Mechanistically, the PI3K-AKT-mammalian target of the rapamycin (mTOR) pathway, a key driver of tumor progression, was shown to play a causal role in prostate tumorigenesis through the up-regulation of pyruvate kinase isoenzyme type M2 (PKM2), the rate-limiting enzyme catalyzing the final reaction of the glycolytic pathway [151,152]. Mutations of the tumor suppressor p53, frequently occurring in advanced prostate cancers, were reported to trigger the Warburg effect. Moreover, deletions of the tumor suppressor PTEN, often observed in aggressive prostate tumors, were demonstrated to correlate with an increased expression of hexokinase 2 (HK2), the initial enzyme of glycolysis, catalyzing the phosphorylation of glucose by ATP to glucose-6-P through the AKT-mTOR pathway [149,153]. Taken together, these observations support that PTEN and p53 tumor suppressors, together with the PI3K-AKT-mTOR pathway, are essential drivers of the Warburg effect to maintain a sufficient energy and metabolites supply for PCa growth and progression [154]. In addition, it has been demonstrated that, in prostatic carcinoma cell lines, the hypoxic conditions of the tumor microenvironment trigger the expression of SUMO1/sentrin-specific peptidase 1 (SENP1) that in turn interacts with HIF1α to promote the Warburg effect and sustain cell proliferation [155]. In line with these data, Sun and coworkers recently reported that HK2 and HIF1α are highly expressed in PCa tissues and their expression correlates with tumor growth and metastasis [156]. 

There is also consistent evidence that an association exists between obesity and the risk of PCa growth [157]. A deleterious bidirectional cross-talk between PCa cells and adipocytes in their microenvironment has been demonstrated [158]. Specifically, it has been reported that PCa cells educate neighboring adipocytes towards a lipolytic phenotype, resulting in free glycerol production and secretion; adipocyte-derived glycerol is then uptaken by PCa cells to enter and fuel the glycolytic pathway [159]. Moreover, adipocyte conditioning of PCa cells leads to an increased expression of glycolytic genes, resulting in lactate production and OXPHOS inhibition [159,160]. In line with these observations, we recently reported that adipocyte-released extracellular vesicles significantly decrease the sensitivity of PCa cells to the chemotherapeutic drug docetaxel and this effect is associated with an AKT/HIF-1α axis-related Warburg effect, which is characterized by enhanced glucose consumption, lactate release and ATP production [161]. 

To confirm that PCa cells undergo dynamic metabolic changes at each stage of tumor development, Vayalil and Landar introduced the “mitochondrial oncobioenergetic index (MOBI)” (i.e., the mathematical representation of the oncobioenergetic features of a tumor cell). In PCa cells with progressive malignant behaviors, they demonstrated that MOBI values (representative of OXPHOS activity) are high in premalignant prostate cells and significantly decrease with increasing malignancy [133].

Taken together, these observations support that PCa cells reprogram their metabolism towards the aerobic glycolysis (the Warburg effect) in the context of tumor progression. However, contrasting results, demonstrating that the high levels of OXPHOS activity observed in primary tumors still persists during the progression of the pathology towards its metastatic stage, have also been reported in the literature [162,163,164,165]. 

Galbraith and coworkers recently demonstrated an association between peroxisome proliferator-activated receptor gamma (PPARG) expression and metastatic features in PCa. By means of in vitro and in vivo studies, these authors could show that, in PCa cells, PPARG overexpression induces AKT3 expression leading to increased mitochondrial biogenesis and ATP production, finally fueling tumor cell epithelial-to-mesenchymal transition (EMT) and metastatic behavior [166]. Pyruvate dehydrogenase complex (PDC) is the multi-protein complex that catalyzes the conversion of pyruvate to acetyl-CoA, thus fostering mitochondrial activity. It was reported that, in PCa cells, knockout of the major subunit of PDC (PDHA1) is accompanied by lower levels of the TCA cycle activity, resulting in impaired OXPHOS activity and growth of these cells when xenografted in nude mice [163]. Pyruvate kinase isozyme 2 (PKM2) has been shown to be highly expressed in many types of cancer cells, including PCa cells. Interestingly, in these cells, PKM2 has been reported to be also deeply involved in glucose metabolism (OXPHOS activity) and to mediate proliferation, metastatic behavior and acquisition of stem cell properties [167,168,169]. Mitochondrial pyruvate carrier (MPC) is the hetero-dimeric complex (formed by MPC1 and MPC2) responsible for the import of pyruvate into the mitochondria where it is converted to acetyl-CoA and then further enters the TCA cycle to fuel the OXPHOS machinery. MPC2 expression was found to correlate with tumor aggressiveness in PCa specimens [170,171]. Transcriptional enhanced associate domain 4 (TEAD4) is a transcription factor previously shown to be involved in the regulation of the expression of mitochondrial genes involved in the OXPHOS pathways [172]. TEAD4 is expressed in PCa cells, and its expression has been reported to be critical in increasing OXPHOS activity. In a recent paper, Chen and coworkers reported that TEAD4 expression is epigenetically regulated by the semi-essential amino acid arginine to modulate OXPHOS functions in hormone-refractory PCa cells [173]. 

Interestingly, elevated OXPHOS and mitochondrial mass have been observed in the aggressive stem cell subpopulation of different tumors, including PCa [174]. Sotgia and coworkers proposed the development of a “mitochondrial based oncology platform” for specifically targeting CSC metabolism [175,176]. In line with this observation, metformin has been proposed as an effective anticancer agent based on its ability to specifically target OXPHOS and ATP production in prostate CSCs [177]. On the other hand, impaired mitochondrial OXPHOS and upregulated glycolysis were observed in these cells [178]. Thus, the presence of exacerbated OXPHOS in PCa stem cells still remains a controversial issue.

Given the crucial role of the tumor-stroma cross-talk in shaping cancer cell metabolism, Ippolito et al. investigated how CAFs might regulate mitochondrial dynamics in PCa cells. They found that tumor-associated CAFs significantly enhance mitochondrial respiration, mediated by a lactate shuttle, and favor mitochondria transfer in PCa cells, thus promoting their malignant behavior [46]. In line with these results, Grupp et al. demonstrated that a high mitochondria content in PCa specimens correlates positively with PCa progression and represents an effective predictor of a poor clinical prognosis and outcome [132]. Last but not least, a switch from glycolysis to OXPHOS activity has been observed in PCa cells undergoing the development of resistance to standard therapies (i.e., enzalutamide, docetaxel) [179].

Based on these observations, it is now accepted that targeting both glycolysis and mitochondrial OXPHOS pathway might represent an effective therapeutic strategy for advanced, metastatic and drug-resistant PCa [180].

**Figure 1:**
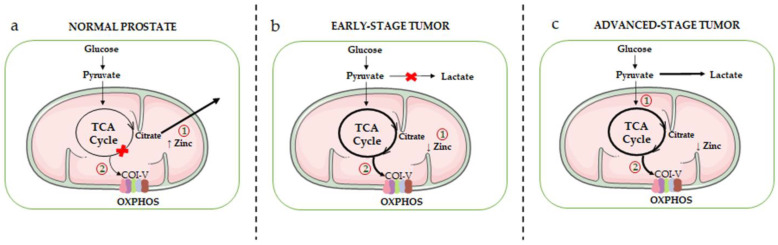
Schematic overview of the mitochondrial metabolic rewiring occurring in prostate epithelial cells during the different stages of cancer progression. (**a**) Healthy prostate epithelial cells accumulate high levels of zinc (due to the overexpression of its transporter ZIP1), resulting in the inhibition of mitochondrial m-aconitase, the key enzyme responsible for the citrate-isocitrate conversion in the TCA cycle (1). This inhibition ultimately leads to the truncation of the TCA cycle and to citrate accumulation and secretion. As a result, normal prostate epithelial cells are characterized by an inefficient OXPHOS machinery (complexes I-V, COI-V) (2). (**b**) In the early stage of tumor progression, intracellular zinc levels are significantly reduced (due to a decreased expression of its transporter) (1); this leads to the reactivation of m-aconitase, restoring the citrate-isocitrate conversion, and consequently of the TCA cycle and OXPHOS metabolic pathways (2). (**c**) In the advanced stage of tumor progression, PCa cells exhibit the Warburg effect, an active aerobic glycolysis accompanied by high levels of lactate production (1). However, it is now well established that a high activity of the TCA cycle/OXPHOS pathways still persists in these cells, and it is even exacerbated in PCa stem cells as well as in drug-resistant PCa cells (2).

A schematic overview of the metabolic rewiring occurring in prostate epithelial cells during the different stages of cancer progression is given in Figure 1.

#### 2.1.2. The AR-Mitochondria Axis

ADT still represents the therapy of choice for early-stage, androgen-dependent PCa. However, most patients progress towards the aggressive CRPC stage characterized by a high rate of cell proliferation, invasiveness and metastatic behavior; interestingly, in most CRPC patients (about 80%) a reactivation of the androgen receptor (AR) has been observed. The persistent activity of AR in this stage has been shown to involve different mechanisms, including AR gene amplification, AR mutations, AR gene alternative splicing, generating different receptor splice variants and intratumoral synthesis of androgens. Since PCa progression is also associated with a peculiar metabolic reprogramming, as discussed above, it has been postulated that AR might be a master regulator of PCa cell metabolism. In line with this hypothesis, genomic, transcriptomic and metabolomic functional studies pointed out that AR regulates different metabolic pathways, including glucose uptake (through the induction of different glucose transporters), glycolysis, TCA cycle, mitochondrial biogenesis and respiration, de novo lipid synthesis and fatty acid β-oxidation [143,170,181,182,183,184]. 

Different intracellular signaling pathways were reported to be involved in this AR-driven metabolic reprogramming. Importantly, AR activation was demonstrated to induce mTOR translocation into the nucleus where it binds to the promoter regions of metabolic genes (such as HK2), thereby regulating their expression; accordingly, inhibition of the mTOR pathway resulted in impaired glycolytic activities and reduced proliferation in PCa cells [183]. In AR-expressing PCa cells, it was shown that androgens promote the activity of the AMPK/PGC1α signaling cascade, leading to increased glycolytic rates, mitochondrial biogenesis, OXPHOS, intracellular ATP levels and cell growth [182]. 

An additional interesting mediator of the AR metabolic activity is MPC (specifically the MPC2 isoform), reported to be highly expressed in AR-positive PCa cells (both hormone-dependent and CRPC cells) but almost absent in AR-negative PCa cells and to play a pivotal role in supporting a functional TCA cycle. Bader and coworkers demonstrated that MPC is transcriptionally upregulated by AR in PCa cells, and its inhibition impairs O_2_ consumption, TCA cycle metabolite levels and oxidative phosphorylation, thus halting cell proliferation. Moreover, these authors could show that targeting MPC with the MPC inhibitors UK5099 and MSDC0160 (this inhibitor is orally administered and clinically viable) results in the suppression of the growth of AR-expressing, but not AR-negative, PCa cells in in vitro and in vivo studies [143,170,185]. In line with these results, it has been reported that, in androgen-sensitive and CRPC cells, activation of the AR signaling upregulates the expression of DRP1 (the mediator of the mitochondrial fission) to induce the formation of the VDAC/MPC2 complex and thereby the pyruvate transport into the mitochondria and sustains mitochondrial metabolic pathways, such as OXPHOS. 

Ultimately, DRP1 overexpression was observed to positively correlate with cancer cell proliferation and survival in different conditions of metabolic stress, such as oxidative stress and hypoxia; moreover, high DRP1 expression levels in patients with CRPC were found to be suggestive of poor prognosis [171]. Interestingly, by means of in vitro and in vivo models, Xu et al. recently reported a loss of MPC expression during the progression of PCa cells towards the most aggressive and AR-negative neuroendocrine phenotype (NEPC); in these models, MPC loss induces the expression of PKM2 that, in turn, translocates into the nucleus to regulate the expression of EMT markers [186]. 

On the other hand, it has been shown that knockout of KDM4B, a transcriptional activator of AR, in CRPC cells reduces proliferation and triggers a metabolic switch towards OXPHOS [187]. Bajpai and coworkers found that, in experimental models of AR overexpression in AR-negative PCa cells, AR localizes to mitochondria where it decreases mitochondrial DNA (mtDNA) content and impairs OXPHOS activity [188]. 

Taken together, these data suggest that, even if most of the published data support that in the PCa models in which it is expressed and active (i.e., AR-driven hormone-sensitive and castration-resistant PCa) AR plays a pivotal role as a master regulator of the cancer cell metabolic reprogramming towards mitochondrial respiration, further studies are needed to definitely assess this issue.

#### 2.1.3. mtDNA Mutations

So far, most studies addressing the relevance of genetic mutations in PCa development have been focused on the nuclear genome. Mitochondria, maternally inherited organelles, are deeply involved in the process of tumorigenesis by orchestrating metabolic and energy production pathways, ROS signaling and apoptosis [8,57,189]. Thus, dissecting the mitochondrial genome is currently considered an essential step to obtain a complete view of the genetic alteration profile in PCas. 

The majority of the proteins of the four ETC complexes (COI-IV) involved in OXPHOS are encoded by nuclear DNA; however, 13 proteins in these complexes are encoded by mtDNA, the small circular DNA molecule found inside mitochondria. The mtDNA is characterized by a high mutation rate, mainly linked to high levels of ETC-derived ROS and a low efficient DNA repair system in these organelles [190]. Mutations of mtDNA have been found in different types of human cancers, including PCa [162,191,192,193,194,195], although their functional role still needs to be fully elucidated. Gomez-Zaera and coworkers analyzed the presence of mtDNA sequence variants in human PCa tissues; they reported that the most frequent variants were present in the following genes: mt-RNR2, encoding the large 16S mitochondrial ribosomal RNA (rRNA) subunit; mt-D-loop (displacement loop, control sites for the expression of the mitochondrial genome); and mt-ND4, encoding the protein NADH dehydrogenase 4, part of the COI of the ETC pathway [196].

The analysis of somatic mutations in tumor tissues from PCa patients pointed out their presence in genes coding for rRNA (mt-RNR1 and mt-RNR2), transfer RNA (tRNA) and the protein-coding gene mitochondrially encoded ATP synthase membrane subunit 6 (mt-ATP6), that encodes the ATP synthase F_o_ subunit 6 (or subunit/chain A). Moreover, somatic mutations in the entire mitochondrial genome were found to be associated with high PSA levels in PCa patients [197]. By using a yeast model organism, it was shown that the mutation mt-ATP6-P136S specifically found in PCa tissues positively correlates with tumor progression and may be involved in cancer cell escape from apoptosis [198]. Hopkins et al. reported that mutations in mitochondrial rRNA, tRNA as well as in the protein-coding genes mt-ATP6, mt-ND1, mt-ND2 and mitochondrially encoded cytochrome c oxidase I (mt-CO1), component of the complex IV, cytochrome c oxidase, the last enzyme in the mitochondrial electron transport chain which drives OXPHOS, are frequent in PCa tissues and are drivers of PCa aggressive behavior [195]. In line with these observations, mutations in genes encoding for proteins of the mitochondrial complex I (mt-NDs) were reported to be frequent in high grade PCa tissues and to be associated with a reduced activity of the NADH dehydrogenase pathway and an increased, compensatory, activity of the succinate-using FADH2 pathway [199]. Interestingly, Sun and coworkers demonstrated that the presence of a mutant mt-CO1 gene results in the resistance of PCa cells to the pro-death activity of simvastatin [200].

Taken together, these data support that in PCa cells undergoing stressful conditions, activation of the ROS signaling might have a deleterious effect on mtDNA driving mutations at the level of genes coding for proteins deeply involved in the metabolic rewiring. 

### 2.2. Mitochondrial Dynamics 

It is now well established that alterations of the mitochondrial structural dynamics (biogenesis, fusion, fission and mitophagy) are deeply involved in the different steps of cancer growth, progression and development of drug resistance [16,80,82,201,202]. However, current data on the role of the mitochondrial structural alterations in PCa are still scanty. 

PGC1α is the well-recognized master regulator of mitochondrial biogenesis [203]; it is also involved in the control of the mitochondrial fusion/fission balance by promoting fusion, through the activation of MFN1 and 2, and impairing DRP1 expression, through the binding to its promoter region [204]. The expression of this gene, together with the mitochondrial number, was found to be upregulated in tumors, including PCa, of African American cancer patients known to be exposed to a higher risk of cancer and mortality compared to European American patients [205]. PGC1α has been observed to be highly expressed in PCa cells harboring either deletion or mutation of the classic tumor suppressor protein p53, and its expression positively correlates with cancer cell metastatic behavior [206]. It has been demonstrated that, in CRPC PC3 cells, overexpression of p53 decreases the expression and activity (i.e., nuclear localization) of PGC-1α, thereby leading to a reduced mitochondrial mass and a significant change in the expression levels of genes and proteins involved in the fusion/fission balance [207]. More recently, Galbraith and coworkers reported that, in PCa cells, the activity of PGC1α is also regulated by the PPARG/AKT3 axis. Specifically, these authors found that, in CRPC cells, overexpression of the transcription factor PPARG induces the expression of the AKT3 kinase that, in turn, triggers the nuclear localization of PGC1α, thereby driving mitochondrial biogenesis and ATP production which may fuel the metastatic behavior of tumor cells [166]. 

Burch et al. investigated the expression of mitochondrial biogenesis and bioenergetics genes in PCa cells and tissues. They reported that 47 genes involved in mitochondrial biogenesis, fusion/fission and energy metabolism are differentially expressed in malignant vs. non-malignant PCa cells. Specifically, the mitochondrial carrier uncoupling protein 2 (UCP2) gene, involved in energy homeostasis linking mitochondrial metabolism and redox (ROS detoxification), was observed to be overexpressed in malignant PCa cells as well as in clinical PCa specimens [208]. 

Alterations of the fusion/fission balance have also shown to be deeply involved in tumorigenesis, although the data so far available on this issue in PCa cells are still limited. Generally, it is accepted that mitochondria fission, the division of mitochondria in smaller organelles, is a typical feature of cells undergoing apoptosis; moreover, this process foresees the translocation of the cytoplasmic DRP1 protein to the mitochondria where it interacts with its receptor FIS1. In PCa cells, it has been reported that the overload of Ca^2+^ in mitochondria triggers the interaction of DRP1 with FIS1, thereby leading to mitochondrial fragmentation and enhanced cell response to pro-apoptotic agents [209]. In line with these observations, we observed that, in CRPC cells, mitochondrial Ca^2+^ and ROS overload triggers mitochondrial fission and mitophagy to mediate the pro-death (apoptotic, paraptotic) activities of natural anticancer compounds [78]. Moreover, enhanced mitochondrial fusion, together with mutations of the complex I mtDNA, were found to be associated with PCa progression, as evaluated in cancer cell lines as well as in mice and human tissue samples [210].

On the other hand, mitochondrial fission has been recently reported to play a key role in the maintenance of stemness features in prostate CSCs. Specifically, Civenni and coworkers focused their attention on bromodomain and extra-terminal domain (BET) proteins, such as bromodomain containing 4 (BRD4), well known as epigenetic modifiers of gene transcription. These authors showed that the DRP1 receptor, and fission factor, MFF is upregulated in hormone-refractory human prostate tumors as well as in prostate CSCs. Moreover, they could show that BRD4 acts as a key driver of MFF transcription and, therefore, of mitochondrial fission, which is an essential biological event for the survival and self-renewal of CSCs; accordingly, they observed that the inhibition of the BRD4 activity and of the subsequent MFF transcription results in the accumulation of dysfunctional mitochondria and, consequently, in the acquisition of the senescent phenotype in these cells. Thus, mitochondrial fission is a crucial process for the maintenance of the self-renewal and tumorigenic potential of the CSC subpopulation in prostate tumors. The authors conclude that pharmacological inhibition of BRD4 may represent a promising effective therapeutic strategy for PCa patients [211,212]. 

Mitophagy is the intracellular removal of mitochondria by autophagy. It has been shown both to promote cancer cell survival and drug resistance through the degradation of damaged mitochondria and to prevent tumor progression mediating the pro-death activity of different anticancer drugs. Thus, its specific role in regulating the balance between cell survival vs. cell death is still a matter of debate in most tumor types, including PCa [105,106,213,214,215,216]. Han and coworkers reported that pharmacological targeting of the androgen/AR axis promotes apoptosis of PCa cells by triggering the mitophagic pathway [217]. It is well known that the oncogenic epidermal growth factor receptor (EGFR) is overexpressed in different types of tumors to exert its peculiar pro-survival effects. It has been shown that, in PCa cells, downregulation of EGFR expression, but not its tyrosine kinase activity, results in cell death mediated by mitophagy through the activation of the AKT/mTOR [218]. In line with these observations, it has been reported that elevated levels of the mitophagy inhibitor leucine-rich pentatricopeptide repeat motif-containing protein (LRPPRC) positively correlate with poor prognosis and shorter survival in PCa patients, supporting a protective effect of mitophagy against PCa growth [219]. On the other hand, Balvan et al. demonstrated that, in CRPC PC3 cells, activation of mitophagy (degradation of mitochondria upon oxidative stress) represents a crucial mechanism of protection and resistance against pro-oxidant conditions [220].

In summary, mounting evidence supports that mitochondrial dynamics are involved in prostate carcinogenesis; however, further research is needed to definitely assess their role in the growth and development of PCa.

## 3. Targeting Mitochondrial Reprogramming and Dynamics in PCa

The data discussed so far support that mitochondrial metabolic reprogramming and structural dynamics represent promising molecular targets for novel and effective therapeutic strategies for PCa patients. Actually, several “mitochondrial metabolism-interfering agents” are currently being investigated for their anticancer activity in different tumors, including PCa (Table 1).

Docetaxel in combination with ADT therapy as a neoadjuvant intervention before radical prostatectomy in patients with localized, high-risk PCa is now well recognized as an effective strategy to increase overall and progression-free survival. To dissect the molecular mechanisms underlying the beneficial effects of this therapeutic intervention, Qu and coworkers recently investigated the effects of neoadjuvant docetaxel in combination with a GnRH analog on different metabolic pathways in cancer tissues. They reported that the neoadjuvant therapy induces a significant downregulation of metabolic pathways, such as the TCA cycle and lipid synthesis; a decrease of the GSH/GSSG ratio, supporting a reduced oxidative stress condition, was also observed. Glutamine was downregulated in the treatment group, supporting that the energy supply was significantly reduced in these patients as the consequence of a reduced glutamate production and impairment of the TCA cycle [221]. 

Metformin, an FDA-approved biguanide hypoglycemic compound, is the first-line drug for the treatment of type 2 diabetes [222,223]. It is administered orally and is well tolerated, being associated with a low incidence of side effects [224]. Recently, several studies have pointed out that metformin exerts a significant antitumor activity in different types of cancer cells, including PCa cells, both in vitro and in preclinical xenograft models [225,226,227,228,229]. In line with these data, epidemiologic studies evidenced a decrease in the incidence of cancer in metformin-treated diabetic patients [230,231,232]. In PCa cells, this compound has been shown to decrease cell viability, induce cell cycle arrest and promote apoptosis through modulation of different intracellular signaling pathways, including glucose metabolism [233,234,235,236]. Specifically, metformin was reported to interfere with the IGF-1 (insulin-like growth factor-1), the VEGF (vascular-endothelial growth factor) and the androgen receptor signaling pathways [236,237,238,239,240,241] as well as increase ROS production, causing a rapid imbalance in cellular redox homeostasis and causing cell death [242]. Focusing on cell glucose metabolism, it was found to induce the activation (i.e., phosphorylation) of the energy sensor AMPK thus inhibiting downstream mTOR activity, increasing glucose uptake, lactate production, glycolytic rate and, more importantly, inhibiting O_2_ consumption and the complex I of the mitochondrial ETC, thereby impairing the OXPHOS pathway [229,236,241,243,244,245]. Thus, in PCa cells, this drug decreases ATP levels, triggering energy deficiency and reduced lipogenesis [246]. Metformin was also found to target the viability of CSCs deeply involved in drug resistance and cancer relapse through the inhibition of mitochondrial bioenergetics and the subsequent compensative increase of the glycolytic pathway, and by triggering mitochondrial ROS production ultimately leading to apoptosis [177,243,247,248]. In line with these data, Ippolito and coworkers demonstrated that CRPC cells made resistant to docetaxel acquire an invasive phenotype associated both with a decreased glycolytic rate and with an overactivation of oxidative phosphorylation; by targeting mitochondrial complex I, metformin suppresses the proliferative and invasive behavior of drug-resistant cells [31]. Moreover, by addressing the issue of the stroma/tumor interplay in PCa, Giannoni et al. observed that CAFs induce an aggressive phenotype in PCa cells by triggering PKM2 nuclear translocation and consequent metabolic reprogramming towards oxidative phosphorylation; pharmacological targeting of PKM2 (by DASA-58) and OXPHOS activity (by metformin) significantly counteracts CAF-induced PCa cell growth in vitro and in vivo [167]. Similar observations were reported with other antidiabetic drugs, such as the metformin analog phenformin and the SGTL2 (sodium-glucose transporter 2) inhibitor canagliflozin [52,249]. The antitumor activity of metformin and phenformin in PCa cells was also reported to be potentiated by co-treating the cells with drugs shown to impair different aspects of cell metabolism, such as 2-deoxyglucose, an inhibitor of HK2 activity and inducer of intracellular ATP depletion [250]; gossypol, an inhibitor of ALDH (cytosolic aldehyde dehydrogenase) and, therefore, of cytosolic NADH production, which selectively reduces electron supply for ATP production [52]; and salicylate, the aspirin metabolite known to activate the AMPK pathway [251]. Recent clinical studies investigated the activity of metformin, either alone or in combination with other anticancer compounds, in PCa patients. Metformin was reported to be associated with improved disease-specific survival in PCa patients, with treatment starting at tumor diagnosis or after prostatectomy [252,253,254]; moreover, it was shown to improve the outcome in PCa patients co-treated with ADT [255,256]. However, contrasting results were also reported. Cui and coworkers, by performing a systematic literature search and meta-analysis, reported the lack of association between metformin and the risk of PCa in diabetic patients [257]. Moreover, this drug also failed to improve the efficacy of standard treatment regimens, such as bicalutamide and docetaxel, in overweight and in metastatic castration-resistant PCa patients, respectively [258,259]. Several clinical trials aimed to definitely dissect the antitumor activity of metformin and its analog phenformin, either alone or in combination with common treatments, are currently being conducted in PCa patients (https://www.clinicaltrials.gov/) (accessed on 21 December 2022).

Many other compounds were reported to counteract PCa growth by targeting the mitochondrial OXPHOS pathway through the inhibition of the ETC complexes. In CRPC DU145 cells, the class III antiarrhythmic drug amiodarone, currently available in the clinical setting, was found to impair the activity of complex I and II, thereby suppressing ATP production and potentiating the antitumor activity of standard therapies (docetaxel and cisplatin) [260]. PCa cells made resistant to the AR antagonist enzalutamide (ENZA) switch from glycolysis to OXPHOS. Basu et al. demonstrated that the complex I inhibitor IACS-010759 exerts a significant antiproliferative activity in ENZA-resistant PCa cells, when compared to non-resistant cells; similar results were observed with the mitochondrial glutaminase inhibitor CB-839, which acts by decreasing the glutamate supply to the TCA cycle [179]. 

Mitochondrial ATP synthase, or complex V, is the fifth OXPHOS complex, responsible for the synthesis of ATP. High levels of ATP synthase were observed in different tumors, including PCa. In cancer cells, ATP synthase has also been shown to translocate from the mitochondria to the cell membrane where it contributes to many processes, such as tumorigenesis, angiogenesis and metastasis. Several ATP synthase synthetic inhibitors were reported to suppress cancer growth and are currently undergoing clinical investigation [261]. Specifically, Elbehairi et al. reported that, in PCa cells, the Pd(II) complex of Gboxin analog-chitooligosaccharides conjugate (Pd(II)COS@GbA) effectively impairs ATP synthase expression and activity, leading to the suppression of ATP production, and triggers mitochondrial fragmentation (fission) by increasing the expression levels of the fission protein DRP1 while decreasing those of the fusion protein OPA1 [262]. 

It is now accepted that some antibiotics can induce death in cancer cells by impairing mitochondrial functions and, based on these properties, are being used as anticancer drugs [263]. According to the endosymbiotic theory, mitochondria are organelles that derive from prokaryotic aerobic cells that were engulfed by eukaryotic cells during their evolution process. In line with this theory, mitochondria contain a DNA different from that of the nucleus and their own protein biosynthesis machinery. Lisanti and coworkers demonstrated that in different types of cancer cells, including PCa cells, and specifically in their stem cell counterpart, some antibiotics (i.e., azithromycin, doxycycline, tigecycline, pyrvinium pamoate, chloramphenicol) impair mitochondrial respiration by binding to mitoribosomes thus inhibiting protein synthesis and by impairing mitochondrial biogenesis, OXPHOS and ATP production [264,265,266,267]. These authors proposed the efficacy of the mitochondrial-based oncology platform (MITO-ONC-RX), a panel of FDA-approved antibiotics, to reduce mitochondrial mass and OXPHOS for the eradication of CSCs in different tumors, including PCa [176]. In line with these observations, the antimicrobial peptide Buforin IIb, in combination with 2-deoxyglucose, displays a synergistic toxic effect on CRPC cells, in vitro and in mouse xenograft tumors. Mechanistically, the anticancer effects of the combination treatment were mediated by a decrease of lactate production (i.e., inhibition of glycolysis) and intracellular ATP levels [268]. Further studies, focusing on the metabolic reprogramming-mediated anticancer activity of antibiotics, either alone or in combination with standard therapies, in PCa patients are warranted.

Different natural compounds were also reported to be endowed with significant anticancer activity in PCa, based on their antioxidant effects but also mediated by a rewiring of mitochondrial functional and structural dynamics. 

Tocotrienols (TTs; α-, β-, δ- and γ-TT) are hydrophobic compounds derived from vitamin E and present in some plant sources, such as annatto (*Bixa orellana*) seeds, rice bran and palm oil [269,270]. These compounds have been widely reported to be endowed with different beneficial health activities in the prevention or treatment of chronic diseases, including osteoporosis, cardiovascular and neurodegenerative diseases [271,272,273,274,275,276,277]. Specifically, over the last couple of decades, TTs have also emerged as potential anticancer agents, based on their antiproliferative, proapoptotic, antimetastatic and antiangiogenic activities in several types of cancer cells, including PCa cells [278,279,280,281,282,283,284,285,286,287]. We recently reported that, in CRPC cells (DU145 and PC3), δ-TT triggers apoptosis, involving ER stress and autophagy, as well as paraptosis, a non-canonical cell death mechanism. [288]. Mechanistically, we could demonstrate that this compound inhibits glucose uptake and lactate production in PTEN-deficient LNCaP and PC3 PCa cells by specifically decreasing HK2 expression, and that it synergizes with metformin in inducing cell death. δ-TT also impairs mitochondrial respiration through the downregulation of the expression of OXPOHS complexes (complex I, II and IV), O_2_ consumption and ATP production. These energy-depleting effects were associated with the induction of mitochondrial fission, which is associated with the Ca^2+^- and ROS-mediated mitophagic pathway. Thus, in CRPC cells, δ-TT exerts a significant anticancer activity by impairing the mitochondrial functional and structural dynamics and dysregulating mitochondrial Ca^2+^-ROS homeostasis [78,289].

**Table 1:** Anticancer drugs targeting mitochondrial functional rewiring and structural dynamics in PCa cells.

Drug	Therapy Class	Effects	References
Docetaxel + ADT	Taxane drugs + GnRH analogs	Inhibition of TCA cycle; decrease of the GSH/GSSG ratio; downregulation of glutamine	[221]
Metformin, phenformin canagliflozin	Antidiabetic (type 2 diabetes) drugs	Inhibition of ETC complex I; decreased O_2_ consumption; AMPK activation; suppression of the OXPHOS pathway and ATP production; upregulation of ROS levels	[31,52,167,177,229,236,242,243,244,245,246,247,248,249,250,251]
Amiodarone	Class III antiarrythmic drugs	Inhibition of ETC complex I and II; suppression of ATP production	[260]
IACS-010759, CB-839	Small molecule inhibitors	Inhibition of ETC complex I; inhibition of mitochondrial glutaminase and glutamate supply to the TCA cycle; decreased OXPHOS pathway	[179]
Pd(II)COS@GbA	Pd(II) anticancer complexes	Inhibition of ETC complex V (ATP synthase); suppression of ATP production; mitochondrial fission	[262]
Doxycycline, azithromycin, tigecycline, pyrvinium pamoate, chloramphenicol	Antibiotics	Inhibition of the OXPHOS pathway and mitochondrial respiration; dysregulations of ETC complexes; suppression of ATP production; impairment of mitochondrial biogenesis	[176,264,265,266,267]
Buforin IIb	Antimicrobial peptides	Inhibition of glycolysis and ATP production	[268]
Vitamin E-derived tocotrienols, curcumin, resveratrol, triterpenoids, sulforaphane, phenethyl isothiocyanate (PEITC), silibinin, atpenin A5 analogs, jasmonates, alternol	Natural antioxidants	Reduced O_2_ consumption, ETC protein (complexes COI-V) levels and activity; suppression of the OXPHOS pathway and ATP production; decreased glycolytic pathway; AMPK activation; altered Ca^2+^/ROS homeostasis; mitochondrial fission, mitophagy	[78,180,261,289,290,291,292,293,294,295,296,297,298,299,300,301,302,303,304,305,306]

Curcumin, also called diferuloylmethane, is the main natural curcuminoid present in the Indian spice turmeric. It has been shown to exert a growth-suppressive activity, which is mainly based on its ability to induce oxidative stress in different types of cancers, including PCa [307,308,309]. Recent articles highlighted the role of mitochondrial reprogramming as a novel molecular mechanism underlying the antitumor activity of this polyphenol. Specifically, Ossikbayeva and coworkers recently reported that curcumin, either alone or in association with carnosic acid, inhibits cell proliferation in CRPC cells by inducing cellcycle arrest. These anticancer effects were associated with a dysfunction of mitochondrial respiration, as evidenced by a decrease of O_2_ consumption and of the activities of all the complexes (COI-IV) of the ETC, and were counteracted by cotreating the cells with the mTOR inhibitor rapamycin [290]. In line with these data, curcumin was recently shown to impair mitochondrial phosphorylation in PCa cells by specifically targeting mitochondrial ATP synthase, thus suppressing ATP production [261,291]. 

Resveratrol, a polyphenolic compound found in different vegetables such as grape, berry and peanut fruits, has been shown to have anti-inflammatory, antioxidant, immunomodulatory and antitumor activities [310,311,312,313]. In cancer cells, this polyphenol was widely shown to induce apoptosis by promoting ROS overproduction [292]. By dissecting the biochemical mechanisms underlying the resveratrol anticancer activity, Rodriguez-Enriquez et al. reported that, in cervix cancer HeLa cells, resveratrol inhibits cell proliferation by significantly decreasing both glycolysis and OXPHOS while triggering ROS production and mitophagy [293]. However, contrasting results were reported in PCa cells; specifically, it was observed in CRPC PC3 cells that resveratrol significantly impairs cell growth and that this is coincident with increased mitochondrial biogenesis, fusion and respiration [314]. Thus, further studies are needed to definitely highlight the involvement of mitochondrial metabolic reprogramming and dynamics in the anticancer activity of resveratrol in PCa.

Triterpenoids are plant-derived isopentenyl pyrophosphate oligomers; so far, more than 20,000 triterpenoids have been identified [315]. These compounds have been widely reported to have pharmacological properties, including anti-inflammatory, antioxidant, antipyretic and cardioprotective activities; moreover, several studies revealed their ability to induce cell cycle arrest, cell cytotoxicity/apoptosis, anti-invasion and autophagy [316,317]. In line with these observations, in vitro and in vivo studies pointed out that a great number of triterpenoids exhibit a potent antitumor activity against various types of cancer cells, including PCa cells, through the regulation of different molecular pathways [318,319,320,321,322,323,324]. Among the various molecular mechanisms, they were reported to exert their anticancer activity by impacting tumor metabolism [325,326,327]. Specifically, in PCa cells, triterpenoids were shown to reduce glucose uptake, aerobic glycolysis, O_2_ consumption and intracellular ATP levels and to induce a sustained activation of AMPK and consequent suppression of mTOR signaling [180,294,295,296,297,298].

1,4-Naphthoquinones represent the most important class of quinones found to be endowed with anti-inflammatory, antiallergic, antibacterial, radical scavenging and, importantly, anticancer activity [328]. Dyshlovoy and coworkers recently reported that glucose-conjugated 1,4-naphthoquinones significantly impair CRPC PC3 cell proliferation through the inhibition of pro-survival and drug resistance mechanisms, such as AR signaling and autophagy. These compounds also inhibit glucose uptake, disrupt mitochondrial OXPHOS by suppressing the expression of components of the five complexes of the ETC (COI-COV) and trigger a mitochondrial ROS overload [299].

Sulforaphane is a natural plant isothiocyanate found in many cruciferous vegetables like broccoli, cabbage, cauliflower and kale [329]. In CRPC cells, sulforaphane has been reported to induce mitochondrial apoptosis mediated by an increased expression of the Bax protein. This compound also impairs mitochondrial structural dynamics by inducing mitochondrial biogenesis and fragmentation through the stabilization of the transcription factor Nrf2 (nuclear factor E2-related factor) and the increase of PGC1α expression levels [300]. In line with these data, it was shown that the natural compound PEITC (phenethyl isothiocyanate), another isothiocyanate found in cruciferous vegetables, is a specific inhibitor of complex III that acts by impairing oxidative phosphorylation and ATP synthesis, leading to ROS overproduction and cell death, in PCa cells [301]. 

Silibinin, also known as silybin (both from Silybum, the name of the plant from which it is extracted), is the major active constituent of silymarin, a standardized extract of the milk thistle seeds. It has been shown to induce apoptosis in different cancer cells, both in vitro and in vivo [330,331]. In PC3 cells, silibinin was observed to induce apoptosis by triggering mitochondrial ROS generation and disruption of Ca^2+^ homeostasis, leading to ER stress response [302].

Other natural compounds reported to induce PCa cell death by impairing mitochondrial functional plasticity and dynamics include atpenin A5 analogs (specific inhibitors of complex II activity) [303], jasmonates (inducers of mitochondrial ATP depletion) [304], alternol (inhibitor of TCA cycle, mitochondrial respiration and ATP production) [305] and deguelin (COI inhibitor) [306]. Some of these compounds are presently under investigation in clinical trials aimed to assess their bioavailability, lack of side effects and chemopreventive activity as well as their ability to prevent the progression to metastatic disease in PCa patients (https://www.clinicaltrials.gov/; accessed on 21 December 2022).

## 4. Conclusions

Mitochondria are known to play a pivotal role in crucial biological processes, such as cell metabolism, cell proliferation/death and cell signaling pathways as well as Ca^2+^ and redox homeostasis. In addition, it is now well accepted that these organelles also undergo a peculiar functional and structural rewiring during the process of tumorigenesis to support cancer growth and progression; however, this plasticity seems to occur specifically in the different types of tumor cells and cell contexts. 

Prostate epithelial cells are characterized by a distinctive metabolic reprogramming during the different phases of their transformation from healthy cells to early-stage and, sequentially, to late-stage tumor cells. Specifically, healthy cells accumulate high levels of zinc due to elevated levels of its ZIP1 transporter at the plasma membrane. High intramitochondrial zinc levels inhibit the activity of m-aconitase, the enzyme responsible for the conversion of citrate into isocitrate in the TCA cycle, thus leading to an impairment of its oxidation and to its accumulation and secretion out of the cells. Consequently, healthy epithelial cells are characterized by a truncated TCA cycle and an impaired OXPHOS pathway, which is compensated by an increased glycolytic rate. Moreover, in these cells high intracellular zinc levels were also found to correlate with the mitochondria-mediated intrinsic apoptotic pathway. On the other hand, in early-stage prostate cancer cells the expression of ZIP1 decreases significantly, leading to low intramitochondrial zinc levels. As a consequence, early-stage PCa cells display a reactivation of m-aconitase and citrate oxidation as well as the TCA cycle and OXPHOS pathways, to meet their high demand for energy and building blocks for the synthesis of biomolecules. The low intracellular levels of zinc also allow these cells to avoid apoptosis, thus sustaining cancer cell proliferation. Interestingly, PCa cells undergo an additional mitochondrial metabolic rewiring during their progression toward the late stage of tumorigenesis. The Warburg effect was initially proposed as the main metabolic feature of aggressive/metastatic PCa cells. However, accumulating evidence strongly supports that high levels of the TCA cycle as well as of OXPHOS activity and ATP production are still present in late-stage as well as in drug-resistant PCa cells. Interestingly, it has been shown that both the presence of an active AR axis and mutations in mitochondrial genes are deeply involved in these mechanisms. 

As reported for different types of tumors, distinctive mitochondrial structural dynamics (biogenesis, fusion/fission, mitophagy) have also been observed in PCa cells and specifically in late-stage/CRPC cells. Based on the data discussed here, it has become widely accepted during the last years that mitochondrial metabolic rewiring and dynamics might represent novel molecular markers of PCa growth and progression, as well as interesting targets for novel anticancer therapeutic strategies. Accordingly, it was demonstrated that, in PCa cells, a wide range of drugs, including both synthetic and natural compounds, exert a significant anticancer activity by specifically targeting both the rewired mitochondrial metabolic pathways (O_2_ consumption, TCA cycle, OXPHOS and ATP production) and the altered mitochondrial dynamics. However, most of these results are still derived from in vitro and preclinical studies; thus, clinical trials are urgently required to definitely assess the effectiveness of these compounds in improving treatment options for PCa patients.
